# Supplementary material for: Multimodal foundation model and benchmark for comprehensive retinal OCT image analysis
Source: NPJ Digit Med. 2025 Sep 25;8:576. doi: 10.1038/s41746-025-01852-3 (PMC12462498; doi:10.1038/s41746-025-01852-3)
Supplement: Supplementary file 1 — Supplementary Information [file 41746_2025_1852_MOESM1_ESM.pdf]

Multimodal foundation model and benchmark for comprehensive retinal OCT image analysis

José Morano[1,*], Botond Fazekas[1], Emese Sükei[2], Ronald Fecso[1], Taha Emre[2], Markus Gumpinger[2], Georg Faustmann[1], Marzieh Oghbaie[1], Ursula Schmidt-Erfurth[2], and Hrvoje Bogunović[1,*]

[1]Christian Doppler Laboratory for Artificial Intelligence in Retina, Institute of Artificial Intelligence, Center for Medical Data Science, Medical University of Vienna, Vienna, Austria
[2]OPTIMA Lab, Department of Ophthalmology, Medical University of Vienna, Vienna, Austria
[*]jose.moranosanchez@meduniwien.ac.at, hrvoje.bogunovic@meduniwien.ac.at

Supplementary Information

Supplementary Note 1: Extended results

In the following, we provide the extended tables of the experimental results, showing not only the average performance across all datasets, but also the results for each individual dataset. For the classification tasks, we report the area under the receiver operating characteristic curve (AUROC), average precision (AP), and balanced accuracy (BAcc). For the segmentation tasks, we report the Dice score, intersection over union (IoU), and 95th percentile of the Hausdorff distance (HD95) for all datasets except RETOUCH. For the RETOUCH dataset, we used the official evaluation server of the challenge to compute the metrics, which include the Dice score and absolute volume difference (AVD). To facilitate the comparison of the results, we highlight the best results in bold and underline the second best results. In addition, we have color-coded the best results according to the model. The color code is as follows (excluding models with no best results): **MIRAGE** (blue), **RETFound**[1] (red), **DINOv2**[2] (green), **SL-IN**[3] (black), **nnUNet**[4] (magenta), **MIRAGE–FFT** (cyan), **MultiMAE**[5] (teal), **MAE-OCT** and **MAE-SLO** (salmon), **MIRAGE-Base** (blue-gray), **OCT+Layers** (lime), and **TransUNet** (dark gray). In all cases, the one-tailed Student's t-test was used to assess the statistical significance of the performance difference between the best (in bold) and second best (underlined) models in each dataset. The Wilcoxon signed-rank test was used to assess the statistical significance of the performance differences across all datasets. Statistical significance is indicated by asterisks in the tables: *$p < 0.05$, **$p < 0.01$, ***$p < 0.001$. In those cases where in-house datasets were used for the evaluation, we provide the average performance across both all the datasets and only public datasets (excluding in-house datasets).

Supplementary Table 1: Performance of MIRAGE and state-of-the-art foundation models SL-IN[3], DINOv2[2], and RETFound[1] on the different datasets for the diagnosis and staging of ocular diseases on OCT. All models are based on the ViT-Large architecture and were tuned using linear probing. Statistical significance between the best (bold) and second best (underlined) models in each and across all datasets was assessed using the one-tailed Student's t-test and Wilcoxon signed-rank test, respectively (* $p < 0.05$, ** $p < 0.01$, *** $p < 0.001$).

Dataset	Model	AUROC	AP	BAcc
**Duke iAMD**	SL-IN	97.63 ± 0.23	97.56 ± 0.35	94.49 ± 0.45
	DINOv2	<u>99.13 ± 0.34</u>	<u>99.23 ± 0.30</u>	95.53 ± 0.80
	RETFound	98.24 ± 0.66	98.69 ± 0.50	<u>96.03 ± 1.72</u>
	MIRAGE	**99.52 ± 0.23****	**99.52 ± 0.23****	**96.71 ± 1.42**
**GAMMA**	SL-IN	74.83 ± 1.67	64.02 ± 1.89	54.72 ± 2.72
	DINOv2	71.99 ± 2.62	60.87 ± 3.75	45.83 ± 4.48
	RETFound	<u>82.30 ± 2.27</u>	<u>73.27 ± 3.65</u>	<u>59.17 ± 2.72</u>
	MIRAGE	**87.50 ± 0.64****	**81.00 ± 1.89***	**63.61 ± 4.16***
**Harvard Glaucoma**	SL-IN	75.11 ± 0.90	74.19 ± 0.92	69.32 ± 1.02
	DINOv2	72.29 ± 2.31	72.60 ± 2.48	64.80 ± 1.94
	RETFound	<u>82.14 ± 0.68</u>	<u>81.53 ± 0.71</u>	<u>74.14 ± 1.28</u>
	MIRAGE	**82.75 ± 0.65**	**82.54 ± 0.68**	**76.15 ± 1.25**
**Kermany**	SL-IN	98.65 ± 0.04	95.71 ± 0.12	86.15 ± 0.68
	DINOv2	98.67 ± 0.06	95.87 ± 0.11	<u>86.48 ± 0.39</u>
	RETFound	<u>98.92 ± 0.07</u>	<u>96.50 ± 0.14</u>	85.89 ± 0.51
	MIRAGE	**99.53 ± 0.01*****	**98.39 ± 0.04*****	**91.40 ± 0.25*****
**Noor Eye Hospital**	SL-IN	97.03 ± 0.78	95.06 ± 1.21	84.67 ± 2.67
	DINOv2	<u>97.53 ± 0.67</u>	<u>96.19 ± 0.86</u>	<u>90.00 ± 2.11</u>
	RETFound	95.77 ± 0.87	94.24 ± 0.96	86.00 ± 2.49
	MIRAGE	**98.63 ± 0.40****	**97.69 ± 0.68****	**92.67 ± 3.27**
**OCTDL**	SL-IN	96.86 ± 0.16	91.24 ± 0.21	**79.69 ± 1.39**
	DINOv2	95.22 ± 0.42	88.26 ± 1.06	<u>77.59 ± 1.95</u>
	RETFound	<u>96.96 ± 0.28</u>	<u>91.72 ± 0.66</u>	74.30 ± 1.39
	MIRAGE	**98.27 ± 0.15*****	**93.32 ± 0.27****	76.93 ± 2.94
**OCTID**	SL-IN	98.72 ± 0.11	95.71 ± 0.44	**89.54 ± 0.81**
	DINOv2	98.81 ± 0.20	95.96 ± 0.83	85.04 ± 2.49
	RETFound	<u>98.98 ± 0.28</u>	**96.81 ± 0.65**	<u>88.16 ± 2.04</u>
	MIRAGE	**99.07 ± 0.28**	<u>96.54 ± 0.84</u>	87.53 ± 1.58
**OLIVES**	SL-IN	<u>97.89 ± 0.09</u>	<u>97.48 ± 0.11</u>	<u>95.79 ± 0.54</u>
	DINOv2	94.13 ± 0.23	93.46 ± 0.22	88.64 ± 0.39
	RETFound	**98.05 ± 0.15***	**97.68 ± 0.25**	**95.81 ± 0.27**
	MIRAGE	96.06 ± 0.09	94.36 ± 0.24	93.14 ± 0.27
**OPTIMA9C** (in-house)	SL-IN	97.33 ± 0.07	86.65 ± 0.32	69.63 ± 0.83
	DINOv2	97.44 ± 0.08	86.81 ± 0.14	67.27 ± 1.22
	RETFound	<u>98.62 ± 0.08</u>	<u>91.76 ± 0.32</u>	<u>74.02 ± 0.64</u>
	MIRAGE	**99.01 ± 0.02*****	**93.52 ± 0.16*****	**77.95 ± 0.45*****
Average	SL-IN	92.67 ± 9.51	88.63 ± 11.21	80.44 ± 12.86
	DINOv2	91.69 ± 10.64	87.69 ± 12.23	77.91 ± 15.10
	RETFound	<u>94.44 ± 6.66</u>	<u>91.35 ± 8.15</u>	<u>81.50 ± 11.48</u>
	MIRAGE	**95.59 ± 5.80*****	**92.99 ± 6.39*****	**84.01 ± 10.51*****
Average (public only, excluding in-house)	SL-IN	92.09 ± 9.93	88.87 ± 11.87	81.80 ± 13.02
	DINOv2	90.97 ± 11.07	87.80 ± 12.96	79.24 ± 15.50
	RETFound	<u>93.92 ± 6.89</u>	<u>91.30 ± 8.65</u>	<u>82.44 ± 11.84</u>
	MIRAGE	**95.17 ± 6.02*****	**92.92 ± 6.77*****	**84.77 ± 10.91*****

Supplementary Table 2: Performance of MIRAGE and state-of-the-art foundation models SL-IN[3], DINOv2[2], and RETFound[1] on the different datasets for the diagnosis of ocular diseases on SLO. All models are based on the ViT-Large architecture and were tuned using linear probing. Statistical significance between the best (bold) and second best (underlined) models in each and across all datasets was assessed using the one-tailed Student's t-test and Wilcoxon signed-rank test, respectively (*$p < 0.05$, **$p < 0.01$, ***$p < 0.001$).

Dataset	Model	AUROC	AP	BAcc
**OPTIMA9C**	SL-IN	<u>91.96 ± 0.15</u>	<u>75.99 ± 0.45</u>	<u>52.96 ± 1.42</u>
	DINOv2	91.89 ± 0.18	73.22 ± 0.44	52.67 ± 1.45
	MIRAGE	**93.40 ± 0.14*****	**77.38 ± 0.47***	**55.55 ± 0.45****
**OLIVES**	SL-IN	<u>73.22 ± 2.34</u>	**73.71 ± 2.76**	<u>64.61 ± 2.70</u>
	DINOv2	73.14 ± 3.38	71.55 ± 3.61	58.61 ± 2.12
	MIRAGE	**74.08 ± 0.52**	<u>73.40 ± 0.52</u>	**66.72 ± 1.14**
Average	SL-IN	<u>82.59 ± 9.52</u>	<u>74.85 ± 2.28</u>	<u>58.79 ± 6.21</u>
	DINOv2	82.52 ± 9.68	72.39 ± 2.70	55.64 ± 3.48
	MIRAGE	**83.74 ± 9.67***	**75.39 ± 2.05**	**61.13 ± 5.65****

Supplementary Table 3: Performance of MIRAGE and RETFound[1] models on the first and last scans of the OLIVES dataset (DME/DR discrimination) under linear probing. OLIVES data comes from two clinical studies where patients are treated throughout the process. As shown in the original paper[6], treatment produces meaningful changes in the retina, and causes a domain shift in the data for the same patient, which is reflected in the performance of the models on the last scans. The lower robustness of MIRAGE to this domain shift can be explained by the much lower number of samples from diabetic patients used in the pretraining of the model. In particular, less than 2% of the samples from our VIBES dataset are from diabetic patients, while 85% of the samples from the dataset used to train RETFound are from the Moorfields diabetic image dataset (MEH-MIDAS)[1].

Dataset	Model	AUROC	AP	BAcc
**OLIVES (first scans)**	RETFound	98.80 ± 0.75	98.92 ± 0.67	**95.00 ± 0.00**
	MIRAGE	**100.00 ± 0.00**	**100.00 ± 0.00**	**95.00 ± 0.00**
**OLIVES (last scans)**	RETFound	**94.00 ± 3.63**	**94.71 ± 3.25**	**84.00 ± 5.83**
	MIRAGE	80.20 ± 3.54	82.76 ± 3.43	70.00 ± 4.47

Supplementary Table 4: Performance of MIRAGE and state-of-the-art foundation models SL-IN[3], DINOv2[2], and RETFound[1] on the cross-dataset evaluation setting for OCT diagnosis. All models are based on the ViT-Large architecture and were tuned using linear probing. Statistical significance between the best (bold) and second best (underlined) models in each and across all datasets was assessed using the one-tailed Student's t-test and Wilcoxon signed-rank test, respectively (*$p < 0.05$, **$p < 0.01$, ***$p < 0.001$).

Dataset	Model	AUROC	AP	BAcc
**Noor Eye Hospital** trained on UMN + Duke Srinivasan	SL-IN	<u>90.05 ± 0.99</u>	<u>82.65 ± 1.82</u>	<u>66.72 ± 5.01</u>
	DINOv2	83.59 ± 1.23	73.49 ± 1.96	**66.77 ± 3.80**
	RETFound	81.63 ± 2.97	66.57 ± 4.34	47.39 ± 1.40
	MIRAGE	**94.79 ± 0.74****	**92.27 ± 0.74*****	64.98 ± 1.70
**UMN + Duke Srinivasan** trained on Noor Eye Hospital	SL-IN	<u>86.23 ± 0.72</u>	<u>80.74 ± 0.77</u>	65.01 ± 1.83
	DINOv2	78.76 ± 1.01	72.73 ± 1.06	68.60 ± 1.82
	RETFound	79.63 ± 1.06	72.81 ± 1.53	<u>68.74 ± 2.87</u>
	MIRAGE	**88.59 ± 3.48**	**85.69 ± 4.78**	**79.81 ± 2.37****

Supplementary Table 5: Performance of MIRAGE with different decoders on different downstream segmentation tasks: Segmenter[7], DPT[8], and ConvNeXt[5,9]. In all cases, the encoder is based on the ViT-Base architecture and is kept frozen during tuning. Statistical significance between the best (bold) and second best (underlined) models across all SLO and OCT datasets was assessed using the Wilcoxon signed-rank test. However, no statistical significance was found ($p > 0.05$). MIRAGE performs particularly well with ConvNeXt and DPT decoders, but still offers adequate performance with the more lightweight Segmenter decoder.

Modality	Dataset	Model	Dice	IoU	HD95 ↓
**SLO**	**SGA (lesions)**	Segmenter	71.97 ± 22.70	61.54 ± 23.88	169.34 ± 78.47
		DPT	**77.07 ± 18.89**	**67.64 ± 21.61**	**153.31 ± 57.12**
		ConvNeXt	74.47 ± 22.11	64.79 ± 24.23	161.13 ± 88.94
**OCT**	**Duke DME (layers)**	Segmenter	72.39 ± 4.52	57.53 ± 5.50	19.45 ± 7.29
		DPT	82.68 ± 3.40	70.96 ± 4.83	**8.23 ± 4.23**
		ConvNeXt	**82.86 ± 3.60**	**71.23 ± 5.12**	8.57 ± 4.01
	**Duke DME (lesions)**	Segmenter	62.38 ± 9.26	45.87 ± 10.00	50.32 ± 18.13
		DPT	63.92 ± 9.48	47.54 ± 10.15	**40.46 ± 11.58**
		ConvNeXt	**69.06 ± 9.15**	**53.36 ± 11.00**	49.32 ± 30.38
	**GOALS (layers)**	Segmenter	82.87	71.45	14.20
		DPT	92.01	85.33	4.89
		ConvNeXt	**92.87**	**86.79**	**4.87**
**OCT & SLO**	***Average***	Segmenter	72.40 ± 7.25	59.10 ± 9.17	63.33 ± 62.75
		DPT	78.92 ± 10.17	67.87 ± 13.49	**51.72 ± 60.27**
		ConvNeXt	**79.82 ± 9.00**	**69.04 ± 12.08**	55.97 ± 63.17

Supplementary Table 6: Performance of MIRAGE and state-of-the-art foundation models DINOv2[2], RETFound[1], and MedSAM[10] on the different datasets for the segmentation of retinal lesions and layers. Mean and standard deviation values are calculated across the patients in each dataset, except for the GOALS and RETOUCH datasets, where patient information is not available. All models are based on the ViT-Large architecture, except for MedSAM, which is only available in the ViT-Base version. The models were fine-tuned using both the decoder-only fine-tuning strategy. Statistical significance between the best (bold) and second best (underlined) models in each and across all datasets was assessed using the one-tailed Student's t-test and Wilcoxon signed-rank test, respectively ($^{*}p < 0.05$, $^{**}p < 0.01$, $^{***}p < 0.001$). † RETOUCH results were obtained using the official evaluation server of the challenge, which only includes the Dice score and absolute volume difference (AVD), which is reported in the HD95 column. Only the Dice score is included for the calculation of the average performance across all datasets.

Modality	Dataset	Model	Dice	IoU	HD95 / AVD† ↓
OCT	AROI (layers)	DINOv2	85.60 ± 2.08	75.90 ± 2.99	13.97 ± 9.07
		RETFound	85.23 ± 1.51	75.46 ± 2.04	8.21 ± 2.30
		MedSAM	90.85 ± 2.13	84.01 ± 2.95	7.80 ± 4.99
		MIRAGE	**93.79 ± 0.85*****	**88.67 ± 1.42*****	**3.25 ± 1.43****
	AROI (lesions)	DINOv2	28.82 ± 8.88	19.64 ± 6.66	69.69 ± 32.14
		RETFound	28.09 ± 8.39	19.42 ± 6.32	67.91 ± 34.61
		MedSAM	38.26 ± 8.86	27.96 ± 7.08	46.65 ± 15.64
		MIRAGE	**52.18 ± 16.63****	**40.89 ± 13.70****	**37.56 ± 12.17***
	Duke DME (layers)	DINOv2	76.44 ± 5.90	63.03 ± 7.64	21.38 ± 11.53
		RETFound	75.32 ± 4.80	61.47 ± 6.00	19.60 ± 12.12
		MedSAM	79.06 ± 4.47	66.14 ± 6.05	12.01 ± 5.16
		MIRAGE	**83.02 ± 2.93****	**71.41 ± 4.18****	**9.81 ± 4.77***
	Duke DME (lesions)	DINOv2	52.27 ± 12.63	36.15 ± 11.34	66.96 ± 43.42
		RETFound	51.53 ± 9.53	35.16 ± 8.91	68.51 ± 25.25
		MedSAM	60.21 ± 9.74	43.60 ± 9.58	55.52 ± 27.47
		MIRAGE	**69.72 ± 9.16***	**54.14 ± 11.14***	**42.45 ± 22.88**
	GOALS (layers)	DINOv2	81.28	68.68	41.34
		RETFound	62.79	47.16	121.79
		MedSAM	90.36	82.57	10.38
		MIRAGE	**92.46**	**86.08**	**4.96**
	RETOUCH (lesions)	DINOv2	53.60	-	0.13†
		RETFound	57.32	-	0.11†
		MedSAM	69.40	-	0.06†
		MIRAGE	**79.60**	-	**0.03**†
	Average	DINOv2	63.00 ± 19.99	52.68 ± 21.29	42.67 ± 22.80
		RETFound	60.05 ± 18.15	47.73 ± 19.58	57.20 ± 40.57
		MedSAM	71.36 ± 18.37	60.86 ± 21.98	26.47 ± 20.34
		MIRAGE	**78.46 ± 14.26***	**68.24 ± 18.40***	**19.61 ± 16.87***
**OCT** cross-dataset	**Duke iAMD (layers)** trained on AROI	DINOv2	77.51 ± 8.36	66.17 ± 10.26	60.93 ± 59.86
		RETFound	78.84 ± 7.59	67.56 ± 9.49	53.87 ± 53.22
		MedSAM	75.77 ± 11.54	66.55 ± 12.22	39.98 ± 31.38
		MIRAGE	**91.06 ± 2.43*****	**84.62 ± 3.38*****	**4.62 ± 4.52*****
**SLO**	**SGA (lesions)**	DINOv2	61.34 ± 24.56	49.13 ± 23.88	210.02 ± 85.59
		MedSAM	72.20 ± 24.05	62.22 ± 25.58	182.22 ± 86.21
		MIRAGE	**75.31 ± 22.16*****	**65.81 ± 24.14*****	**164.42 ± 95.35****

Supplementary Table 7: Performance of MIRAGE and state-of-the-art *specialist* segmentation models SwinUNETR-V2[11], MedNeXt[12], TransUNet[13], and nnUNet[4] on the different datasets for the segmentation of retinal lesions and layers. Mean and standard deviation values are calculated across the patients in each dataset, except for the GOALS and RETOUCH datasets, where patient information is not available. MIRAGE was trained using both the decoder-only fine-tuning and full fine-tuning (FFT) strategies. Statistical significance between the best (bold) and second best (underlined) models in each and across all datasets was assessed using the one-tailed Student's t-test and Wilcoxon signed-rank test, respectively (*$p < 0.05$, **$p < 0.01$, ***$p < 0.001$).

Modality	Dataset	Model	Dice	IoU	HD95 / AVD† ↓
OCT	AROI (layers)	SwinUNETR-V2	94.00 ± 1.42	89.06 ± 2.34	3.66 ± 1.82
		MedNeXt	94.07 ± 1.45	89.19 ± 2.29	3.60 ± 1.68
		TransUNet	94.63 ± 1.27	90.10 ± 2.11	2.74 ± 1.18
		nnUNet	**95.05 ± 0.79**	**90.81 ± 1.34**	<u>2.33 ± 0.79</u>
		MIRAGE	93.79 ± 0.85	88.67 ± 1.42	3.25 ± 1.43
		MIRAGE–FFT	<u>95.02 ± 0.76</u>	<u>90.73 ± 1.30</u>	**2.18 ± 0.47**
	AROI (lesions)	SwinUNETR-V2	48.98 ± 13.64	38.20 ± 10.40	41.27 ± 13.17
		MedNeXt	49.70 ± 14.62	39.22 ± 11.92	37.85 ± 16.77
		TransUNet	54.30 ± 16.23	43.50 ± 12.94	**35.82 ± 13.57**
		nnUNet	**62.99 ± 21.28**	**51.87 ± 19.16**	49.65 ± 30.74
		MIRAGE	52.18 ± 16.63	40.89 ± 13.70	<u>37.56 ± 12.17</u>
		MIRAGE–FFT	<u>60.51 ± 18.79</u>	<u>48.74 ± 15.82</u>	42.09 ± 14.84
	Duke DME (layers)	SwinUNETR-V2	81.90 ± 3.10	69.88 ± 4.26	15.14 ± 5.79
		MedNeXt	80.87 ± 3.48	68.44 ± 4.76	12.51 ± 5.73
		TransUNet	81.52 ± 3.25	69.36 ± 4.42	9.81 ± 5.44
		nnUNet	**83.49 ± 2.55**	**72.15 ± 3.53**	<u>9.31 ± 4.06</u>
		MIRAGE	83.02 ± 2.93	71.41 ± 4.18	9.81 ± 4.77
		MIRAGE–FFT	<u>83.44 ± 3.00</u>	<u>72.08 ± 4.20</u>	**7.85 ± 3.54**
	Duke DME (lesions)	SwinUNETR-V2	54.44 ± 11.16	38.04 ± 10.56	111.72 ± 98.36
		MedNeXt	63.69 ± 8.96	47.24 ± 9.85	77.86 ± 53.93
		TransUNet	65.12 ± 10.24	48.97 ± 11.28	51.15 ± 18.93
		nnUNet	<u>66.05 ± 10.70</u>	<u>50.05 ± 11.55</u>	**38.11 ± 12.22**
		MIRAGE	69.72 ± 9.16	54.14 ± 11.14	42.45 ± 22.88
		MIRAGE–FFT	**70.04 ± 7.99**	**54.36 ± 9.66**	<u>38.86 ± 22.73</u>
	GOALS (layers)	SwinUNETR-V2	91.77	84.92	8.41
		MedNeXt	91.55	84.54	6.86
		TransUNet	92.38	85.97	7.04
		nnUNet	**93.04**	**87.08**	**4.13**
		MIRAGE	<u>92.46</u>	<u>86.08</u>	<u>4.96</u>
		MIRAGE–FFT	92.01	85.30	5.32
	RETOUCH (lesions)	SwinUNETR-V2	74.29	-	0.06†
		MedNeXt	<u>77.43</u>	-	0.04†
		TransUNet	76.87	-	0.05†
		nnUNet	72.93	-	0.06†
		MIRAGE	79.60	-	**0.03**†
		MIRAGE–FFT	**79.61**	-	0.04†
	Average	SwinUNETR-V2	74.23 ± 17.26	64.02 ± 22.09	36.04 ± 40.01
		MedNeXt	76.22 ± 15.48	65.73 ± 19.78	27.73 ± 27.80
		TransUNet	77.47 ± 14.28	67.58 ± 18.84	21.31 ± 18.88
		nnUNet	<u>78.92 ± 12.49</u>	**70.39 ± 17.06**	<u>20.71 ± 19.41</u>
		MIRAGE	78.46 ± 14.26	68.24 ± 18.40	19.61 ± 16.87
		MIRAGE–FFT	**80.10 ± 11.98**	<u>70.24 ± 16.52</u>	**19.26 ± 17.45**
OCT cross-dataset	Duke iAMD (layers) trained on AROI	SwinUNETR-V2	46.88 ± 1.72	43.90 ± 2.70	201.81 ± 24.71
		MedNeXt	47.05 ± 1.47	44.05 ± 2.34	203.56 ± 29.73
		TransUNet	46.88 ± 1.59	43.76 ± 2.61	194.12 ± 36.45
		nnUNet	<u>61.21 ± 15.33</u>	<u>55.35 ± 12.98</u>	<u>97.95 ± 60.82</u>
		MIRAGE	91.06 ± 2.43	84.62 ± 3.38	4.62 ± 4.52
		MIRAGE–FFT	**91.29 ± 2.14*****	**84.92 ± 3.15*****	**3.94 ± 2.27*****
SLO	SGA (lesions)	SwinUNETR-V2	74.51 ± 23.51	65.09 ± 24.88	169.03 ± 94.67
		MedNeXt	77.22 ± 19.62	67.87 ± 22.19	157.15 ± 84.21
		TransUNet	**82.12 ± 15.92***	**73.69 ± 18.65***	**127.19 ± 57.98**
		nnUNet	<u>79.41 ± 19.26</u>	<u>71.33 ± 21.25</u>	136.56 ± 74.10
		MIRAGE	75.31 ± 22.16	65.81 ± 24.14	164.42 ± 95.35
		MIRAGE–FFT	79.36 ± 17.07	70.03 ± 19.92	<u>136.16 ± 43.48</u>

Supplementary Table 8: Performance of a ViT-Base model on the downstream classification tasks with linear probing pretrained with and without retinal layer pseudo-labels on our VIBES[14] dataset. Statistical significance between the best (bold) and second best models in each and across all datasets was assessed using the one-tailed Student's t-test and Wilcoxon signed-rank test, respectively (*$p < 0.05$, **$p < 0.01$, ***$p < 0.001$).

Dataset	Modalities	AUROC	AP	BAcc
**Duke iAMD**	OCT	98.89 ± 0.06	99.01 ± 0.05	93.08 ± 0.80
	OCT+Layers	**99.58 ± 0.03*****	**99.60 ± 0.03*****	**95.72 ± 1.03****
**GAMMA**	OCT	81.51 ± 0.18	**73.62 ± 2.34**	52.22 ± 1.67
	OCT+Layers	**84.86 ± 0.36*****	72.98 ± 0.73	**52.50 ± 2.22**
**Harvard Glaucoma**	OCT	76.61 ± 0.95	77.02 ± 0.69	69.67 ± 0.81
	OCT+Layers	**83.92 ± 0.98*****	**83.87 ± 0.87*****	**77.17 ± 1.89****
**Kermany**	OCT	98.64 ± 0.07	95.84 ± 0.22	84.20 ± 0.39
	OCT+Layers	**98.93 ± 0.01*****	**96.66 ± 0.07*****	**87.60 ± 0.42*****
**Noor Eye Hospital**	OCT	97.50 ± 0.75	96.02 ± 1.08	86.00 ± 3.89
	OCT+Layers	**99.60 ± 0.50****	**99.27 ± 0.91****	**94.67 ± 1.63****
**OCTDL**	OCT	96.65 ± 0.63	90.76 ± 1.32	73.96 ± 2.09
	OCT+Layers	**98.41 ± 0.10****	**94.12 ± 0.16****	**80.92 ± 0.78****
**OCTID**	OCT	98.10 ± 0.07	94.95 ± 0.22	84.41 ± 0.32
	OCT+Layers	**98.60 ± 0.04*****	**95.10 ± 0.08**	**85.78 ± 0.46*****
**OLIVES**	OCT	**96.85 ± 0.03****	**95.84 ± 0.09***	**96.67 ± 0.41***
	OCT+Layers	96.28 ± 0.21	94.66 ± 1.08	92.81 ± 2.14
**OPTIMA9C**	OCT	**99.02 ± 0.01*****	**93.51 ± 0.10****	77.68 ± 0.55
	OCT+Layers	98.82 ± 0.04	93.26 ± 0.10	**78.15 ± 0.73**
Average	OCT	93.75 ± 7.99	90.73 ± 8.59	79.77 ± 12.76
	OCT+Layers	**95.44 ± 6.00*****	**92.17 ± 8.07*****	**82.81 ± 12.62*****

Supplementary Table 9: Performance of a ViT-Base model on the downstream segmentation tasks with linear probing pretrained with and without retinal layer pseudo-labels on our VIBES[14] dataset. Statistical significance between the best (bold) and second best models in each and across all datasets was assessed using the one-tailed Student's t-test and Wilcoxon signed-rank test, respectively (*$p < 0.05$, **$p < 0.01$, ***$p < 0.001$).

Modality	Dataset	Model	Dice	IoU	HD95 / AVD† ↓
**OCT**	**AROI (layers)**	OCT	84.12 ± 2.89	73.58 ± 4.03	13.52 ± 4.93
		OCT+Layers	**89.47 ± 1.76*****	**81.65 ± 2.55*****	**9.09 ± 4.25***
	**AROI (lesions)**	OCT	42.54 ± 17.83	30.29 ± 14.28	72.68 ± 40.85
		OCT+Layers	**47.27 ± 18.17**	**34.84 ± 14.65**	**56.10 ± 28.64***
	**Duke DME (layers)**	OCT	72.23 ± 3.81	57.38 ± 4.62	**23.47 ± 11.61**
		OCT+Layers	**72.99 ± 4.15**	**58.37 ± 5.02***	27.88 ± 14.52
	**Duke DME (lesions)**	OCT	**57.42 ± 16.29**	**41.63 ± 15.00**	65.07 ± 41.03
		OCT+Layers	56.73 ± 11.92	40.34 ± 11.12	**52.89 ± 28.38**
	**GOALS (layers)**	OCT	77.45	63.74	29.41
		OCT+Layers	**81.20**	**68.80**	**25.70**
	**RETOUCH (lesions)**	OCT	64.49	-	0.09†
		OCT+Layers	**66.78**	-	**0.06**†
	Average	OCT	66.37 ± 13.68	53.32 ± 15.51	40.83 ± 23.58
		OCT+Layers	**69.07 ± 14.21***	**56.80 ± 17.42**	**34.33 ± 17.73**
**OCT** cross-dataset	**Duke iAMD (layers)** trained on AROI	OCT	55.40 ± 6.90	39.18 ± 6.09	**214.22 ± 19.67*****
		OCT+Layers	**65.91 ± 3.91*****	**49.65 ± 3.86*****	222.92 ± 15.67

Supplementary Table 10: Performance of a ViT-Base model on the downstream classification tasks with linear probing for different pretraining strategies. The strategies include MultiMAE[5], pretrained on ImageNet using multimodal data, MAE-OCT and MAE-SLO, trained using MAE[15] on the OCT or SLO images of our VIBES[14] dataset, respectively, and MIRAGE, our proposed model based on multimodal MAE trained on our multimodal VIBES dataset. Statistical significance between the best (bold) and second best (underlined) models in each and across all datasets was assessed using the one-tailed Student's t-test and Wilcoxon signed-rank test, respectively (*$p < 0.05$, **$p < 0.01$, ***$p < 0.001$).

Tuning modality	Dataset	Model	AUROC	AP	BAcc
OCT	**Duke iAMD**	MultiMAE	94.86 ± 2.41	95.91 ± 1.85	88.08 ± 1.87
		MAE-OCT	98.89 ± 0.06	99.01 ± 0.05	93.08 ± 0.80
		MIRAGE	**99.05 ± 0.09***	**99.18 ± 0.07***	**94.68 ± 1.26***
	**GAMMA**	MultiMAE	74.64 ± 1.40	61.20 ± 1.60	55.83 ± 2.22
		MAE-OCT	81.51 ± 0.18	73.62 ± 2.34	52.22 ± 1.67
		MIRAGE	**85.52 ± 0.23*****	**74.26 ± 0.57**	**58.89 ± 4.27**
	**Harvard Glaucoma**	MultiMAE	72.06 ± 1.43	70.90 ± 1.57	65.63 ± 2.21
		MAE-OCT	76.61 ± 0.95	77.02 ± 0.69	69.67 ± 0.81
		MIRAGE	**78.57 ± 0.18****	**78.95 ± 0.19****	**70.48 ± 0.40***
	**Kermany**	MultiMAE	97.66 ± 0.02	93.22 ± 0.06	79.44 ± 0.93
		MAE-OCT	98.64 ± 0.07	95.84 ± 0.22	84.20 ± 0.39
		MIRAGE	**98.77 ± 0.03***	**96.23 ± 0.09***	**85.84 ± 0.32*****
	**Noor Eye Hospital**	MultiMAE	96.73 ± 0.54	95.81 ± 0.46	89.33 ± 1.33
		MAE-OCT	97.50 ± 0.75	96.02 ± 1.08	86.00 ± 3.89
		MIRAGE	**98.53 ± 0.50**	**97.52 ± 0.95**	**93.33 ± 4.22**
	**OCTDL**	MultiMAE	93.28 ± 0.54	85.56 ± 0.68	67.28 ± 2.10
		MAE-OCT	96.65 ± 0.63	90.76 ± 1.32	73.96 ± 2.09
		MIRAGE	**97.51 ± 0.14***	**92.61 ± 0.32***	**77.83 ± 0.89***
	**OCTID**	MultiMAE	97.15 ± 0.17	89.43 ± 0.50	75.51 ± 0.85
		MAE-OCT	98.10 ± 0.07	94.95 ± 0.22	84.41 ± 0.32
		MIRAGE	**98.81 ± 0.24****	**95.90 ± 0.82***	**85.83 ± 1.41**
	**OLIVES**	MultiMAE	**97.03 ± 0.31**	95.10 ± 1.04	**96.70 ± 0.39**
		MAE-OCT	96.85 ± 0.03	**95.84 ± 0.09**	96.67 ± 0.41
		MIRAGE	95.00 ± 0.13	91.83 ± 0.12	91.11 ± 0.07
	**OPTIMA9C**	MultiMAE	95.64 ± 0.25	81.93 ± 0.68	63.71 ± 2.96
		MAE-OCT	**99.02 ± 0.01****	93.51 ± 0.10	77.68 ± 0.55
		MIRAGE	98.91 ± 0.05	**93.58 ± 0.05**	**78.54 ± 0.10***
	Average	MultiMAE	91.01 ± 9.60	85.45 ± 11.59	75.73 ± 13.06
		MAE-OCT	93.75 ± 7.99	90.73 ± 8.59	79.77 ± 12.76
		MIRAGE	**94.52 ± 6.97*****	**91.12 ± 8.15****	**81.84 ± 11.24*****
SLO	**OPTIMA9C**	MultiMAE	89.88 ± 0.95	70.19 ± 2.22	46.60 ± 2.30
		MAE-SLO	84.77 ± 0.48	59.67 ± 1.02	36.23 ± 1.46
		MIRAGE	**92.83 ± 0.25****	**75.60 ± 0.50****	**51.30 ± 0.69***
	**OLIVES**	MultiMAE	77.17 ± 1.14	76.03 ± 1.21	65.65 ± 1.51
		MAE-SLO	64.57 ± 0.38	62.19 ± 0.49	66.98 ± 0.54
		MIRAGE	**78.49 ± 0.74**	**77.49 ± 0.78**	**71.79 ± 0.98*****
	Average	MultiMAE	83.52 ± 6.44	73.11 ± 3.42	56.13 ± 9.72
		MAE-SLO	74.67 ± 10.11	60.93 ± 1.49	51.61 ± 15.42
		MIRAGE	**85.66 ± 7.19****	**76.54 ± 1.15****	**61.55 ± 10.28****

Supplementary Table 11: Performance of a ViT-Base model on the downstream segmentation tasks with linear probing for different pretraining strategies The strategies include MultiMAE[5], pretrained on ImageNet using multimodal data, MAE-OCT, and MAE-SLO, both trained using MAE[15] on the OCT or SLO images of our VIBES[14] dataset, respectively, and MIRAGE, our proposed model based on multimodal MAE trained on our multimodal VIBES dataset. Statistical significance between the best (bold) and second best (underlined) models in each and across all datasets was assessed using the one-tailed Student's t-test and Wilcoxon signed-rank test, respectively (*$p < 0.05$, **$p < 0.01$, ***$p < 0.001$).

**Modality**	**Dataset**	**Model**	**Dice**	**IoU**	**HD95 / AVD† ↓**
OCT	**AROI (layers)**	MultiMAE	74.01 ± 3.16	60.28 ± 3.93	32.46 ± 13.69
		MAE-OCT	84.12 ± 2.89	73.58 ± 4.03	13.52 ± 4.93
		MIRAGE	**89.73 ± 1.48***	**81.93 ± 2.32***	**8.59 ± 3.52**
	**AROI (lesions)**	MultiMAE	17.72 ± 7.38	11.23 ± 5.02	80.69 ± 28.04
		MAE-OCT	42.54 ± 17.83	30.29 ± 14.28	72.68 ± 40.85
		MIRAGE	**43.84 ± 14.84**	**31.92 ± 11.52**	**69.78 ± 69.78**
	**Duke DME (layers)**	MultiMAE	55.88 ± 8.49	40.97 ± 8.14	38.03 ± 23.22
		MAE-OCT	72.23 ± 3.81	57.38 ± 4.62	23.47 ± 11.61
		MIRAGE	**75.03 ± 3.85***	**60.80 ± 4.91***	**17.44 ± 9.93**
	**Duke DME (lesions)**	MultiMAE	1.29 ± 1.78	0.65 ± 0.91	165.52 ± 77.51
		MAE-OCT	57.42 ± 16.29	41.63 ± 15.00	65.07 ± 41.03
		MIRAGE	**62.68 ± 7.72**	**46.01 ± 8.23**	**43.54 ± 24.19**
	**GOALS (layers)**	MultiMAE	65.30	48.97	85.58
		MAE-OCT	77.45	63.74	**29.41**
		MIRAGE	**80.52**	**68.07**	32.27
	**RETOUCH (lesions)**	MultiMAE	32.34	-	0.16†
		MAE-OCT	64.49	-	0.09†
		MIRAGE	**65.99**	-	**0.07†**
	Average	MultiMAE	41.09 ± 26.13	32.42 ± 22.72	80.46 ± 47.68
		MAE-OCT	66.37 ± 13.68	53.32 ± 15.51	40.83 ± 23.58
		MIRAGE	**69.63 ± 14.60***	**57.75 ± 17.35***	**34.33 ± 21.42**
**OCT** cross-dataset	**Duke iAMD (layers)** trained on AROI	MultiMAE	33.97 ± 11.20	21.32 ± 8.22	226.25 ± 27.87
		MAE-OCT	55.40 ± 6.90	39.18 ± 6.09	**214.22 ± 19.67***
		MIRAGE	**63.32 ± 4.19***	**46.76 ± 4.03***	217.92 ± 16.78
**SLO**	**SGA (lesions)**	MultiMAE	68.69 ± 23.43	57.76 ± 24.21	187.07 ± 88.52
		MAE-SLO	70.63 ± 22.54	59.79 ± 23.36	174.96 ± 78.10
		MIRAGE	**72.24 ± 21.68**	**61.79 ± 23.55**	**166.25 ± 73.90**

Supplementary Table 12: Performance of the ViT-Large and ViT-Base versions of MIRAGE on the downstream classification tasks using linear probing. Statistical significance between the best (bold) and second best models in each and across all datasets was assessed using the one-tailed Student's t-test and Wilcoxon signed-rank test, respectively (*$p < 0.05$, **$p < 0.01$, ***$p < 0.001$).

Modality	Dataset	Model	AUROC	AP	BAcc
OCT	**Duke iAMD**	MIRAGE-B	99.05 ± 0.09	99.18 ± 0.07	94.68 ± 1.26
		MIRAGE-L	**99.52 ± 0.23****	**99.52 ± 0.23****	**96.71 ± 1.42**
	**GAMMA**	MIRAGE-B	85.52 ± 0.23	74.26 ± 0.57	58.89 ± 4.27
		MIRAGE-L	**87.50 ± 0.64****	**81.00 ± 1.89*****	**63.61 ± 4.16**
	**Harvard Glaucoma**	MIRAGE-B	78.57 ± 0.18	78.95 ± 0.19	70.48 ± 0.40
		MIRAGE-L	**82.75 ± 0.65*****	**82.54 ± 0.68*****	**76.15 ± 1.25*****
	**Kermany**	MIRAGE-B	98.77 ± 0.03	96.23 ± 0.09	85.84 ± 0.32
		MIRAGE-L	**99.53 ± 0.01*****	**98.39 ± 0.04*****	**91.40 ± 0.25*****
	**Noor Eye Hospital**	MIRAGE-B	98.53 ± 0.50	97.52 ± 0.95	**93.33 ± 4.22**
		MIRAGE-L	**98.63 ± 0.40**	**97.69 ± 0.68**	92.67 ± 3.27
	**OCTDL**	MIRAGE-B	97.51 ± 0.14	92.61 ± 0.32	**77.83 ± 0.89**
		MIRAGE-L	**98.27 ± 0.15*****	**93.32 ± 0.27****	76.93 ± 2.94
	**OCTID**	MIRAGE-B	98.81 ± 0.24	95.90 ± 0.82	85.83 ± 1.41
		MIRAGE-L	**99.07 ± 0.28**	**96.54 ± 0.84**	**87.53 ± 1.58**
	**OLIVES**	MIRAGE-B	95.00 ± 0.13	91.83 ± 0.12	91.11 ± 0.07
		MIRAGE-L	**96.06 ± 0.09*****	**94.36 ± 0.24*****	**93.14 ± 0.27*****
	**OPTIMA9C** (in-house)	MIRAGE-B	98.91 ± 0.05	**93.58 ± 0.05**	**78.54 ± 0.10***
		MIRAGE-L	**99.01 ± 0.02***	93.52 ± 0.16	77.95 ± 0.45
	Average	MIRAGE-B	94.52 ± 6.97	91.12 ± 8.15	81.84 ± 11.24
		MIRAGE-L	**95.59 ± 5.80*****	**92.99 ± 6.39*****	**84.01 ± 10.51*****
	Average (public only, no in-house)	MIRAGE-B	93.97 ± 7.21	90.81 ± 8.60	82.25 ± 11.86
		MIRAGE-L	**95.17 ± 6.02*****	**92.92 ± 6.77*****	**84.77 ± 10.91*****
SLO	**OPTIMA9C** (in-house)	MIRAGE-B	92.83 ± 0.25	75.60 ± 0.50	51.30 ± 0.69
		MIRAGE-L	**93.40 ± 0.14****	**77.38 ± 0.47*****	**55.55 ± 0.45*****
	**OLIVES**	MIRAGE-B	**78.49 ± 0.74*****	**77.49 ± 0.78*****	**71.79 ± 0.98****
		MIRAGE-L	74.08 ± 0.52	73.40 ± 0.52	66.72 ± 1.14
	Average	MIRAGE-B	**85.66 ± 7.19**	**76.54 ± 1.15**	**61.55 ± 10.28**
		MIRAGE-L	83.74 ± 9.67	75.39 ± 2.05	61.13 ± 5.65

Supplementary Table 13: Performance of the ViT-Large and ViT-Base versions of MIRAGE on the downstream segmentation tasks for OCT and SLO datasets using decoder-only fine-tuning and full fine-tuning (FFT). Mean and standard deviation values are calculated across the patients in each dataset, except for the GOALS and RETOUCH datasets, where patient information is not available. Statistical significance between the best (bold) and second best models in each and across all datasets was assessed using the one-tailed Student's t-test and Wilcoxon signed-rank test, respectively (*$p < 0.05$, **$p < 0.01$, ***$p < 0.001$).

Modality	Dataset	Model	Dice	IoU	HD95 / AVD† ↓
**OCT**	**AROI (layers)**	MIRAGE-B	**94.04 ± 1.18**	**89.12 ± 1.93**	3.39 ± 1.12
		MIRAGE-L	93.79 ± 0.85	88.67 ± 1.42	**3.25 ± 1.43**
	**AROI (lesions)**	MIRAGE-B	49.12 ± 16.58	38.11 ± 13.67	38.64 ± 14.99
		MIRAGE-L	**52.18 ± 16.63**	**40.89 ± 13.70**	**37.56 ± 12.17**
	**Duke DME (layers)**	MIRAGE-B	82.86 ± 3.60	71.23 ± 5.12	**8.57 ± 4.01**
		MIRAGE-L	**83.02 ± 2.93**	**71.41 ± 4.18**	9.81 ± 4.77
	**Duke DME (lesions)**	MIRAGE-B	69.06 ± 9.15	53.36 ± 11.00	49.32 ± 30.38
		MIRAGE-L	**69.72 ± 9.16**	**54.14 ± 11.14**	**42.45 ± 22.88**
	**GOALS (layers)**	MIRAGE-B	**92.87**	**86.79**	**4.87**
		MIRAGE-L	92.46	86.08	4.96
	**RETOUCH (lesions)**	MIRAGE-B	75.87	-	0.06†
		MIRAGE-L	**79.60**	-	**0.03**†
	Average	MIRAGE-B	77.30 ± 15.37	67.72 ± 19.58	20.96 ± 19.17
		MIRAGE-L	**78.46 ± 14.26**	**68.24 ± 18.40**	**19.61 ± 16.87**
**OCT** cross-dataset	**Duke iAMD (layers)** trained on AROI	MIRAGE-B	90.14 ± 3.33	83.30 ± 4.27	12.29 ± 10.04
		MIRAGE-L	**91.06 ± 2.43*****	**84.62 ± 3.38*****	**4.62 ± 4.52*****
**SLO**	**SGA (lesions)**	MIRAGE-B	74.47 ± 22.11	64.79 ± 24.23	**161.13 ± 88.94**
		MIRAGE-L	**75.31 ± 22.16**	**65.81 ± 24.14**	164.42 ± 95.35

Supplementary Note 2: Benchmark datasets

This section provides a brief description of the 16 datasets used in the evaluation benchmark. B-scan dimensions are always in the format [B-scan height (A-scan depth) × B-scan width (# A-scans)] pixels. The number of B-scans per volume is indicated in each case. The field of view (FOV) always refers to the retinal *en-face* view. The datasets are publicly available unless otherwise noted, and links to the datasets are provided.

Classification datasets

In the following, we provide a brief description of the 11 classification datasets used in the evaluation benchmark. For all datasets that provide full OCT volumes, we limited our analysis to the central B-scan, as done in previous work[1]. Examples of images from each dataset and the corresponding ground truth labels are shown in Supplementary Figure 1.

Duke iAMD[16]. The Duke intermediate age-related macular degeneration (Duke iAMD) dataset includes 38,400 SD-OCT B-scans from 269 iAMD patients and 115 healthy subjects centered on a 5 mm diameter at the fovea. The images were acquired using a Bioptigen SD-OCT system (Research Triangle Park, NC) at four different clinics in the USA. Each volume has a retinal FOV of $\sim 6.7 \times 6.7$ mm^2, and consists of 100 B-scans with dimensions of 512×1000 pixels. In addition to the diagnosis, the dataset includes expert-annotated segmentation maps for three key retinal regions: the inner limiting membrane (ILM) to the inner retinal pigment epithelium (RPE) detachment complex (RPEDC), the inner RPEDC to the outer Bruch's membrane (BM), and below the BM. For the classification evaluation, we focused on the detection of iAMD, while for the segmentation evaluation, we focused on the segmentation of all the aforementioned layers. Link: `https://people.duke.edu/~sf59/RPEDC_Ophth_2013_dataset.htm`

Duke Srinivasan[17]. This dataset presented by Srinivasan et al.[17] consists of OCT volumes acquired from 45 patients. The scans were categorized according to the diagnosis of the patients: healthy (15 samples), dry AMD (15), and diabetic macular edema (DME) (15). The scans were acquired using a Spectralis device (Heidelberg Engineering, Heidelberg, Germany) at Duke University, Harvard University, and the University of Michigan, USA. The FOV and OCT dimensions are very heterogeneous; for more information, see Table 1 from the original publication[17]. For the purposes of this study, we focused on the detection of AMD and DME. Link: `https://people.duke.edu/~sf59/Srinivasan_BOE_2014_dataset.htm`

GAMMA[18]. This dataset originates from the Glaucoma grAding from Multi-Modality imAges (GAMMA) challenge[18], held in conjunction with the International Conference on Medical Image Computing and Computer-Assisted Intervention (MICCAI) 2023. It was provided by the Sun Yat-sen Ophthalmic Center, China, and contains pairs of color fundus and OCT images from randomly selected subjects with and without glaucoma. In particular, the public dataset includes 100 samples from the same number of patients. OCT volumes were acquired using a Topcon DRI OCT Triton device (Topcon, Tokyo, Japan). All volumes are centered on the macula with a FOV of 3×3 mm. Each volume has 25 B-scans with dimensions of $992 \times 512 \times$ pixels. The dataset also includes glaucoma stages, foveal coordinates, and cup and optic disc segmentation masks. There are 26 samples with early glaucoma, 24 with moderate or advanced glaucoma, and 50 normal samples. In this study, we focus on the detection and staging of glaucoma. Link: `https://gamma.grand-challenge.org/)`

Harvard Glaucoma[19]. This dataset from the Mass Eye and Ear of Harvard Medical School, USA, consists of 1 000 samples from 1 000 patients. Each sample includes the diagnostic label

Supplementary Figure 1: Example images from classification datasets. OCT and/or SLO images are shown for each dataset along with the corresponding ground truth labels.

(a) Duke iAMD, iAMD

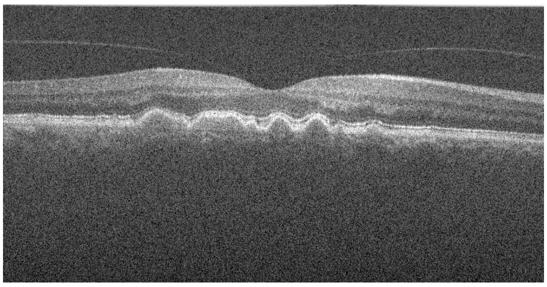

(b) Duke Srinivasan, AMD

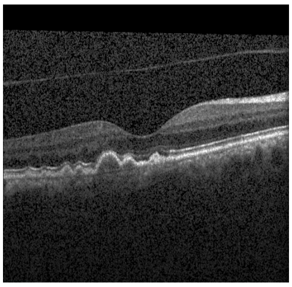

(c) GAMMA, Early glaucoma

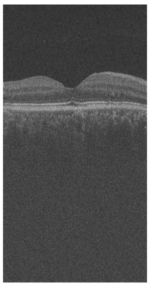

(d) Harvard Glaucoma, Glaucoma

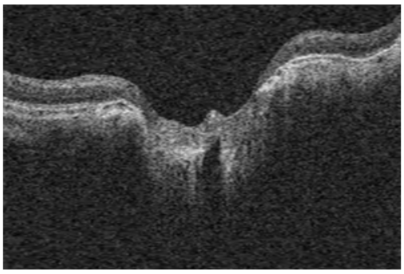

(e) Kermany, CNV

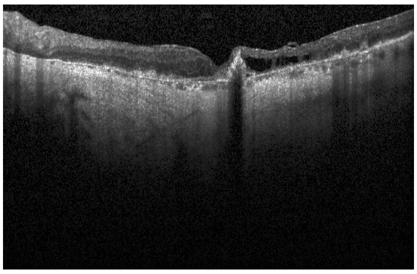

(f) Noor Eye Hospital, Control

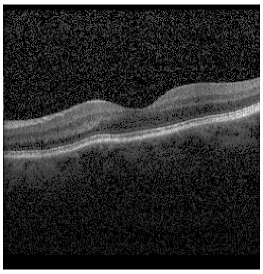

(g) OCTDL, RVO

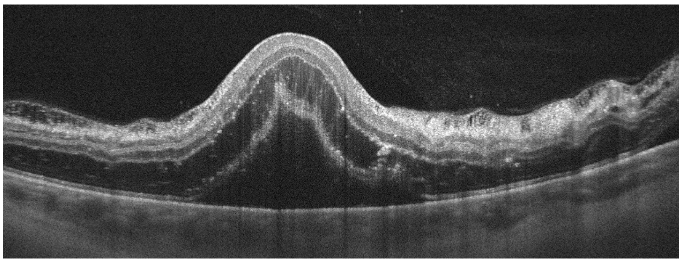

(h) OCTID, DR

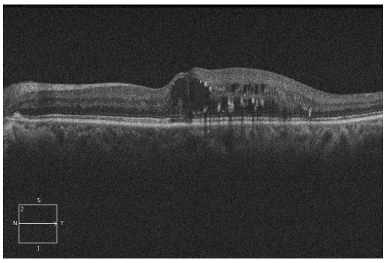

(i) OLIVES, DME

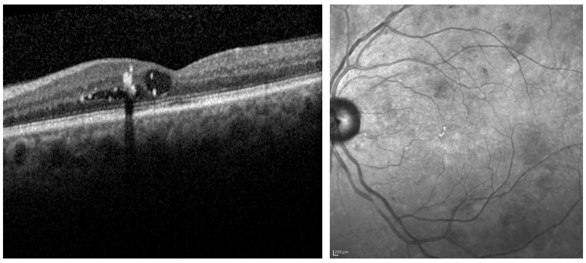

(j) UMN, DME

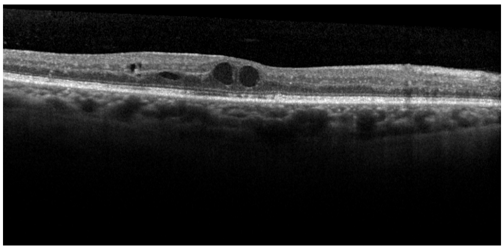

(k) OPTIMA9C, Stargardt disease

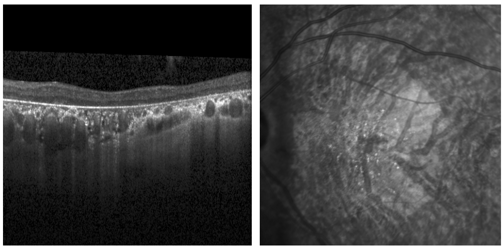

indicating the presence or absence of glaucoma (557 and 443, respectively), an OCT volume centered on the optic nerve head, a retinal nerve fiber layer thickness (RNFLT) map, and other demographic and clinical information such as visual field test results and patient age. The OCT volumes and RNFLT maps were acquired using a Cirrus (Carl Zeiss Meditec, Jena, Germany) device. Each volume has a FOV of $6 \times 6\text{mm}^2$ and 200 B-scans of 200×300 pixels. Link: `https://github.com/Harvard-Ophthalmology-AI-Lab/Harvard-GDP`

Kermany[20,21]. This dataset, presented by Kermany et al.[20,21], consists of 109 309 OCT and 5 862 chest X-ray images. The OCT images, from 4 686 patients, were acquired using an Spectralis device at different clinics in California, USA, Shanghai, China, and Beijing, China. Each sample was then assigned to one of the following four classes: choroidal neovascularization (CNV) (37 455 samples), DME (11 598), drusen (8 866), and control (51 390). The FOVs and dimensions of the OCT scans vary considerably across the dataset[20]. Link: `https://data.mendeley.com/datasets/rscbjbr9sj/2`

Noor Eye Hospital[22]. This dataset from the Noor Eye Hospital in Tehran, Iran, consists of 148 OCT volumes from the same number of patients. Every volume is categorized into one of three classes, depending on the diagnosis: control (50 samples), dry AMD (48), and DME (50). Scans are centered on the macula and were acquired using a Spectralis device with different FOVs, resolutions, and number of B-scans. Link: `https://hrabbani.site123.me/available-datasets/dataset-for-oct-classification-50-normal-48-amd-50-dme`

OCTDL[23]. The Optical Coherence Tomography Dataset for Image-Based Deep Learning Methods (OCTDL) is a public dataset from the Ural Federal University Named afer the first president of Russia B. N. Yeltsin, Russia. The dataset consists of 2 064 B-scans from 821 patients labeled based on disease type and retinal pathology. Scans were acquired using an Optovue Avanti RTVue XR (Fremont, CA, USA) device centered on the fovea with different field of views (FOVs) and image resolutions. Specialists classified the images into one of the following classes: control (332 samples), AMD (1 231), DME (147), epiretinal membrane (ERM) (155), retinal artery occlusion (RAO) (22), retinal vein occlusion (RVO) (101), and vitreomacular interface disease (VID) (76). Link: `https://data.mendeley.com/datasets/sncdhf53xc/4`

OCTID[24]. The Optical Coherence Tomography Image Database (OCTID) is a public dataset of OCT B-scans from the Sankara Nethralaya (SN) Eye Hospital, India. The images were acquired using a Cirrus HD-OCT machine with a raster scan protocol with a 2 mm scan length and an image resolution of 512×1024 pixels. Images were labeled based on the diagnosis of retinal clinical experts at the SN hospital. Specifically, the selected 572 B-scans were categorized into the following classes: control (206 samples), macular hole (MH) (102), AMD (55), central serous retinopathy (CSR) (102), and diabetic retinopathy (DR) (107). In each volumetric scan, a fovea-centered image was selected by an experienced clinical optometrist. The images were then resized to 500x750 pixels. Link: `https://borealisdata.ca/dataverse/OCTID`

OLIVES[6]. The Ophthalmic Labels for Investigating Visual Eye Semantics (OLIVES) is a longitudinal dataset consisting of 78 189 OCT B-scans (1590 volumes) and scanning laser ophthalmoscopy (SLO) (referred to as near-infrared fundus images) from 96 patients with DR or DME. The OLIVES dataset is derived from the PRIME[25] and TREX-DME[26] clinical studies run at the Retina Consultants of Texas, USA. Every volume has around 49 B-scans, with an image resolution of 496×504 pixels. It includes 16 biomarker labels, 4 clinical labels, and a disease diagnosis for either DME (931 samples) or DR (659 samples). The images were acquired using Spectralis devices. Link: `https://zenodo.org/records/7105232`

UMN[27]. The University of Minnesota (UMN) dataset, collected by the University of Minnesota Ophthalmology Clinic, USA, consists of 54 OCT volumes from 30 patients with DME and 24 patients with AMD. All scans were acquired using a Spectralis device centered on the macula. Each volume contains 25 B-scans with an image resolution of $496 \times 1\,024$ pixels. Link: `https://people.ece.umn.edu/users/parhi/.DATA/`

OPTIMA9C[28]. This private dataset comes from the imaging data collection at OPTIMA Lab, Medical University of Vienna, Austria. The dataset contains 4 205 treatment-naive OCT volumes and their corresponding SLO images from 3 652 patients. The OCT volumes are baseline scans from randomized, multicenter clinical trials and include data from several manufacturers: Spectralis, Cirrus, and Triton. The dataset consists of nine classes based on clinical study data: iAMD (clinical studies NCT01790802 and NCT00891735, Observational study 1[29]); three types of CNV (NCT02307682, NCT01780935, NCT01972789); geographic atrophy (GA) (NCT02503332, Observational study 2[30]); RVO (NCT01599650, NCT01535261); DME (NCT01331681, NCT01627249); Stargardt disease (Observational study 3[31]); and healthy samples (NCT03465124 and the fellow eyes from the clinical studies NCT00891735, NCT01780935, NCT01948830, NCT01599650, and NCT01535261). Thus, the classes and the number of samples per class are as follows: control (183), RVO (763), Stargardt (130), DME (1 091), iAMD (1 128), GA (452), CNV1 (99), CNV2 (83), and CNV3 (276). OPTIMA9C is notable for its variation in spatial resolution and disease severity, making it more challenging and unique than other datasets. Image widths range from 200 to 1536 pixels, with heights varying from 480 to 1024 pixels, while the number of slices per volume ranges from 25 to 261 slices, with an average of 81. For privacy reasons, the dataset cannot be made publicly available.

Segmentation datasets

The segmentation datasets used in the evaluation benchmark are briefly described below. In contrast to the classification datasets, all the B-scans in the segmentation datasets are used for training and evaluating the models. This was done following standard practices in the literature, where layer and lesion segmentation tasks are usually performed B-scan-wise, and not on the full volumes[32–34]. Examples of images from each dataset and the corresponding segmentation labels are shown in Supplementary Figure 2.

AROI[35]. The Annotated Retinal Optical coherence tomography Images (AROI) dataset comprises 3 200 B-scans collected from 25 patients diagnosed with neovascular AMD. Macular SD-OCT volumes were acquired with the Zeiss Cirrus HD OCT 4000 device at the Sestre milosrdnice University Hospital Center, Croatia. Each OCT volume consisted of 128 B-scans with a resolution of 1024×512 pixels (pixel size $1.96 \times 11.74 \mu$m). Retinal fluids and layers were annotated for 1 136 B-scans. In our study, one of the OCT volumes was discarded (`patient14`) due to inconsistencies in the annotations, resulting in 3 072 B-scans from 24 patients. Of the fluids, the following were annotated: pigment epithelium detachment (PED), subretinal fluid and subretinal hyperreflective material (both labeled as SRF), and intraretinal fluid (IRF) (named *cyst* in the dataset). Four layer boundaries were also annotated: internal limiting membrane (ILM), inner plexiform layer/inner nuclear layer (IPL/INL), retinal pigment epithelium (RPE), and Bruch's membrane (BM). Translating the annotations from boundaries to layers results in the following labels: ILM–IPL/INL, IPL/INL–RPE, RPE–BM, below the BM. Link: `https://ipg.fer.hr/ipg/resources/oct_image_database`

Duke DME[36]. The Duke Diabetic Macular Edema (Duke DME) dataset, provided by Duke University, USA, consists of 110 annotated OCT B-scan images from 10 patients with severe DME. Each patient features 11 B-scans centered on the fovea with dimensions of 496×536 pixels.

Supplementary Figure 2: Example images from segmentation datasets. OCT and/or SLO images are shown for each dataset along with their corresponding segmentation masks.

(a) AROI

■ background, ■ ILM–IPL/INL, ■ IPL/INL–RPE, ■ RPE–BM, ■ below BM, □ PED, ■ SRF

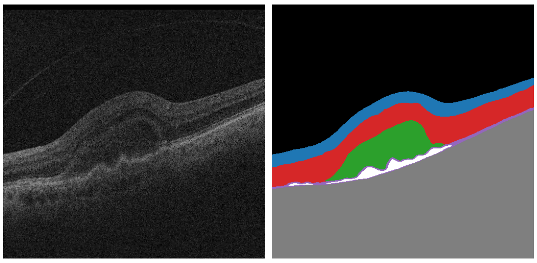

(b) Duke DME

■ background, ■ ILM, ■ RNFL, ■ IPL, ■ INL, ■ OPL, ■ ISM, ■ OS, ■ below BM, □ fluid

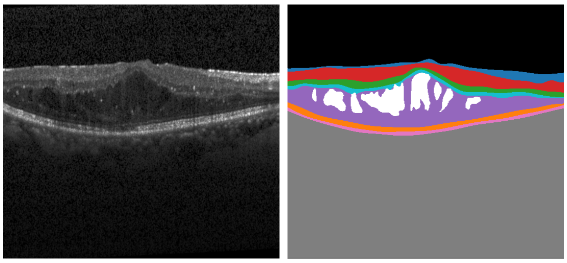

(c) Duke iAMD

■ unlabeled, ■ ILM–Inner RPEDC, ■ Inner RPEDC–BM, ■ below BM

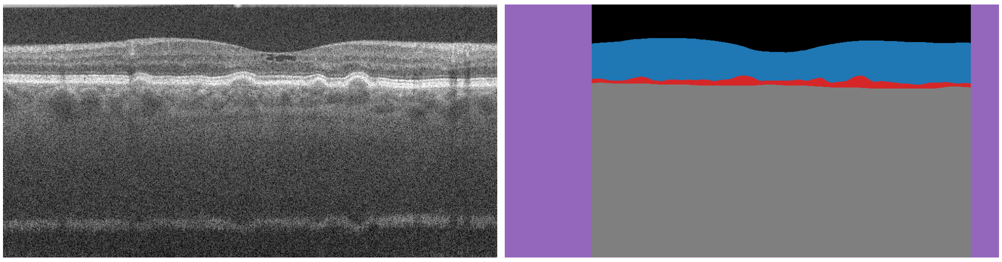

(d) GOALS

■ background, ■ RNFL, ■ GCIPL, ■ choroid

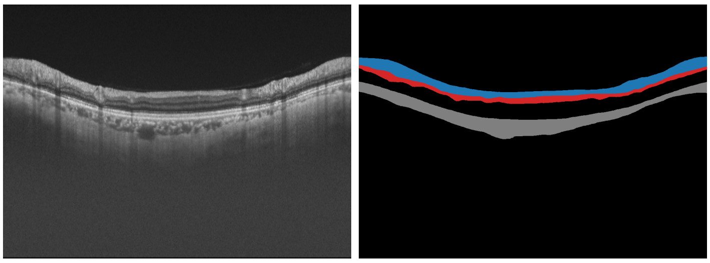

(e) RETOUCH

■ background, ■ IRF, ■ SRF, □ PED

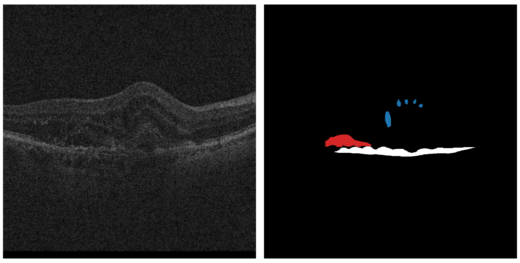

(f) SGA

■ background, □ GA

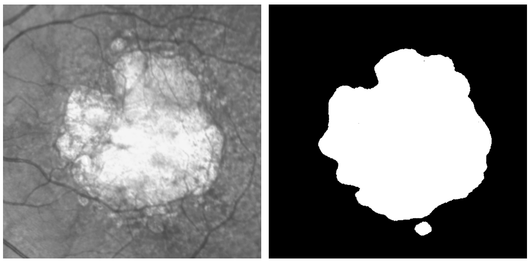

Scans were acquired using a Spectralis device. The dataset includes detailed annotations of eight retinal layer boundaries: ILM, retinal nerve fiber layer (RNFL), ganglion cell layer (GCL), IPL, INL, outer plexiform layer (OPL), outer nuclear layer (ONL), inner segments of photoreceptors (ISM), outer segments of photoreceptors (OS), and RPE. It also includes annotations for fluid regions. Link: `https://people.duke.edu/~sf59/Chiu_BOE_2014_dataset.htm`

Duke iAMD[16]. See the description in the Classification Datasets section above.

GOALS[37]. The Glaucoma OCT Analysis and Layer Segmentation (GOALS) dataset is part of the GOALS Challenge, held in conjunction with MICCAI 2022. The full dataset comprises 300 circumpapillary OCT B-scans randomly selected from glaucoma study cohorts collected at the Zhongshan Ophthalmic Center, China. However, only the 100 B-scans from the training subset were publicly available at the time of preparing the evaluation benchmark, so we used this subset in our study. The number of patients is unknown, as no information is provided in the dataset description and no anonymized patient identifiers are included in the data. FOV information is not provided either. All scans were acquired using a Topcon DRI Swept Source OCT system. All B-scans have a dimension of 800×1100 pixels. Each B-scan is annotated with the following three layers: RNFL, GCL–IPL (GCIPL), and choroid. Since the dataset is not publicly available at the time of writing, we will upload it to our repository: `https://github.com/j-morano/MIRAGE`.

RETOUCH[38]. The retinal OCT fluid detection and segmentation benchmark and challenge (RETOUCH) dataset originates from the MICCAI 2017 retinal OCT fluid challenge. Annotations and scans came from different clinical centers: Medical University of Vienna, Austria, Erasmus University Medical Center, Netherlands, and Radboud University Medical Center, Netherlands. The dataset includes 70 OCT volumes, with half of the patients diagnosed with macular edema secondary to AMD and the other half with edema secondary to RVO. Each B-scan is labeled with three retinal fluid types: IRF, subretinal fluid (SRF), and PED. The training data consists of volumes from three OCT systems: 24 from Cirrus (Model 5000), 22 from Triton (Models T-1000/T-2000), and 24 from Spectralis. Cirrus scans consist of 128 B-scans of 1024×512 pixels, Triton scans consist of 128 B-scans 512×650 or 512×885 pixels, and Spectralis scans consist of 49 B-scans 512×496 pixels. There is at least one fluid lesion in each volume. For this dataset, the results are obtained using the official evaluation script provided by the challenge organizers. Link: `https://retouch.grand-challenge.org/`

SGA[30]. This dataset for the segmentation of geographic atrophy (SGA) is a private dataset consisting of 965 samples consisting of OCT volumes as well as SLO and fundus autofluorescence (FAF) images. All samples come from 100 patients (184 eyes) diagnosed with GA who were part of a clinical study on natural GA progression conducted at the Medical University of Vienna, Austria[30]. The SLO and FAF images were acquired with a Spectralis device centered on the macula with a FOV of 6×6 mm^2 and a resolution of 1024×1024 pixels. OCT and SLO images were co-registered by the device, while FAF and SLO images were registered with an in-house image registration pipeline based on aligning retinal vessel segmentation[39]. All samples have GA *en face* masks annotated by a retinal expert on FAF images. In this work, we used only the SLO images and the corresponding GA masks. For privacy reasons, the dataset cannot be made publicly available.

Supplementary Note 3: Masking strategy

During pretraining, following MultiMAE[5], we sample the number of non-masked tokens for each modality from a symmetric Dirichlet distribution with a concentration parameter $\alpha = 1$. This value results in a diverse sampling across the different modalities. Below, we provide an analysis of the effect of different values of α on the token distribution across three modalities: OCT, SLO, and Layers.

In Supplementary Figure 3, we show a 3D visualization of the number of non-masked tokens per modality for 2000 samples when using different values of α. In the plot, each point represents a sample, and its coordinates correspond to the number of non-masked tokens for each modality. The number of non-masked tokens per modality out of the fixed total (98) was sampled from a Dirichlet distribution with the specified α value.

When α is set to 0.1, most samples are concentrated near the edges of the simplex, meaning that in most cases nearly all tokens are from a single modality. This leads to frequent unimodal processing, potentially causing the model to ignore multimodal interactions.

For $\alpha = 1$, samples are spread across the entire simplex, indicating that the model is more likely to receive tokens from multiple modalities. However, as shown in the plot, it is still possible for the model to receive tokens from only one or two modalities. In this way, the model can learn to process each modality independently while still leveraging multimodal information.

At $\alpha = 100$, almost all samples are tightly clustered around the center of the simplex, indicating that the number of tokens per modality is roughly equal ($\sim$32 tokens per modality). While this configuration ensures balanced multimodal learning, it may limit the ability of the model to process samples where only one modality is available.

To ensure that the model can effectively leverage the multimodal information while still being able to process each modality independently, we set α to 1, which provides a good balance between modality specialization and multimodal learning.

In our training approach, once the *number* of non-masked tokens per modality has been sampled, the non-masked tokens are sampled uniformly at random without replacement and used as input to the model.

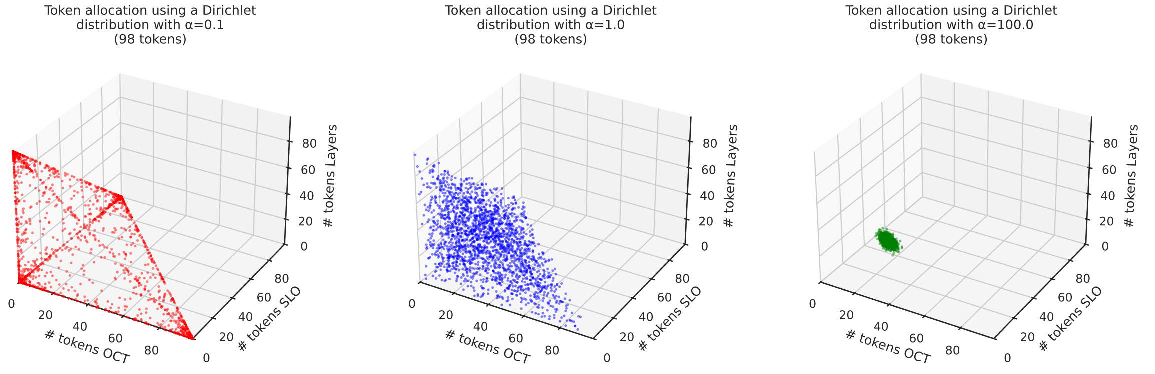

Supplementary Figure 3: 3D scatter plots of token allocations across OCT, SLO, and Layers for different Dirichlet concentration parameters (α = 0.1, 1, and 100). Each point represents a sample, with the coordinates corresponding to the number of non-masked tokens for each modality. A total of 2000 samples are shown for each α value, with the number of total tokens fixed at 98. Lower α values (left) result in sparse, modality-dominant allocations, while higher α values (right) enforce more uniform token distributions.

Supplementary Note 4: Pre-experimental results

To comprehensively assess the effectiveness of the proposed multimodal pretraining approach, we conducted a series of analyses evaluating the quality of the learned representations of MIRAGE without fine-tuning the model on downstream tasks. In particular, we performed three different analyses: training loss, reconstruction visualization, and feature visualization.

Loss curves. To analyze the convergence behavior of the models during pretraining, we show in Supplementary Figure 4 the loss curves for the three training losses (one for each modality) of MIRAGE during the pretraining stage, as well as the total loss and the learning rate. The curves show that the model converges well to values close to zero for all losses, indicating that the model is able to reconstruct the input patches effectively.

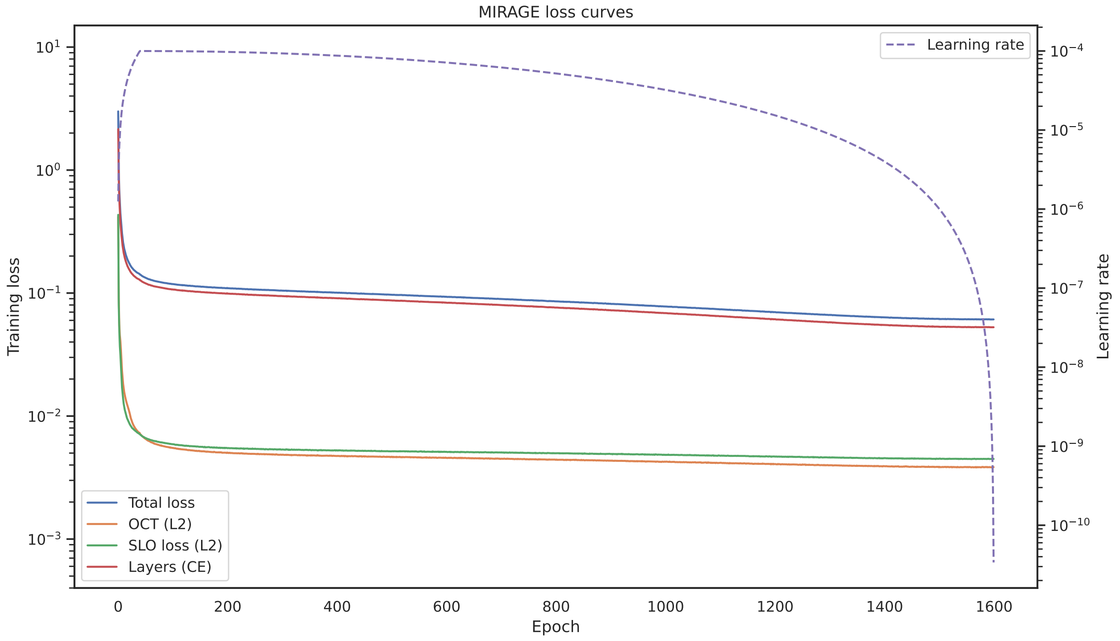

Supplementary Figure 4: Loss curves of MIARGE during pretraining. All training losses for the three modalities (OCT, SLO, and Layers) are significantly minimized during the pretraining stage.

Qualitative reconstruction results. To further validate the effectiveness of our paired multimodal pretraining strategy, we show in Supplementary Figure 5 the predictions of MIRAGE on our internal dataset for different masking ratios of the input modalities. In the figure, it can be observed that the model is able to reconstruct the input patches from the different modalities with relatively high fidelity, even when a large portion of the input modality is masked. For instance, we show that the model is able to predict the segmented layers from the OCT B-scans and the SLO, even when all patches from the input Layers modality are masked. This is also true in the reverse direction, where the model is able to reconstruct the OCT B-scans from the segmented layers and SLO images. Additionally, although the SLO reconstructions are of lower quality compared to OCT and Layers, the model is still able to reconstruct the coarse lesions from the OCT B-scans and the segmented layers. These visualizations suggest that the model effectively learns the relationships between the different modalities during the pretraining phase.

Feature visualization. To provide a qualitative understanding of the representations learned by MIRAGE, we visualize the embeddings produced by it, DINOv2, and RETFound for the

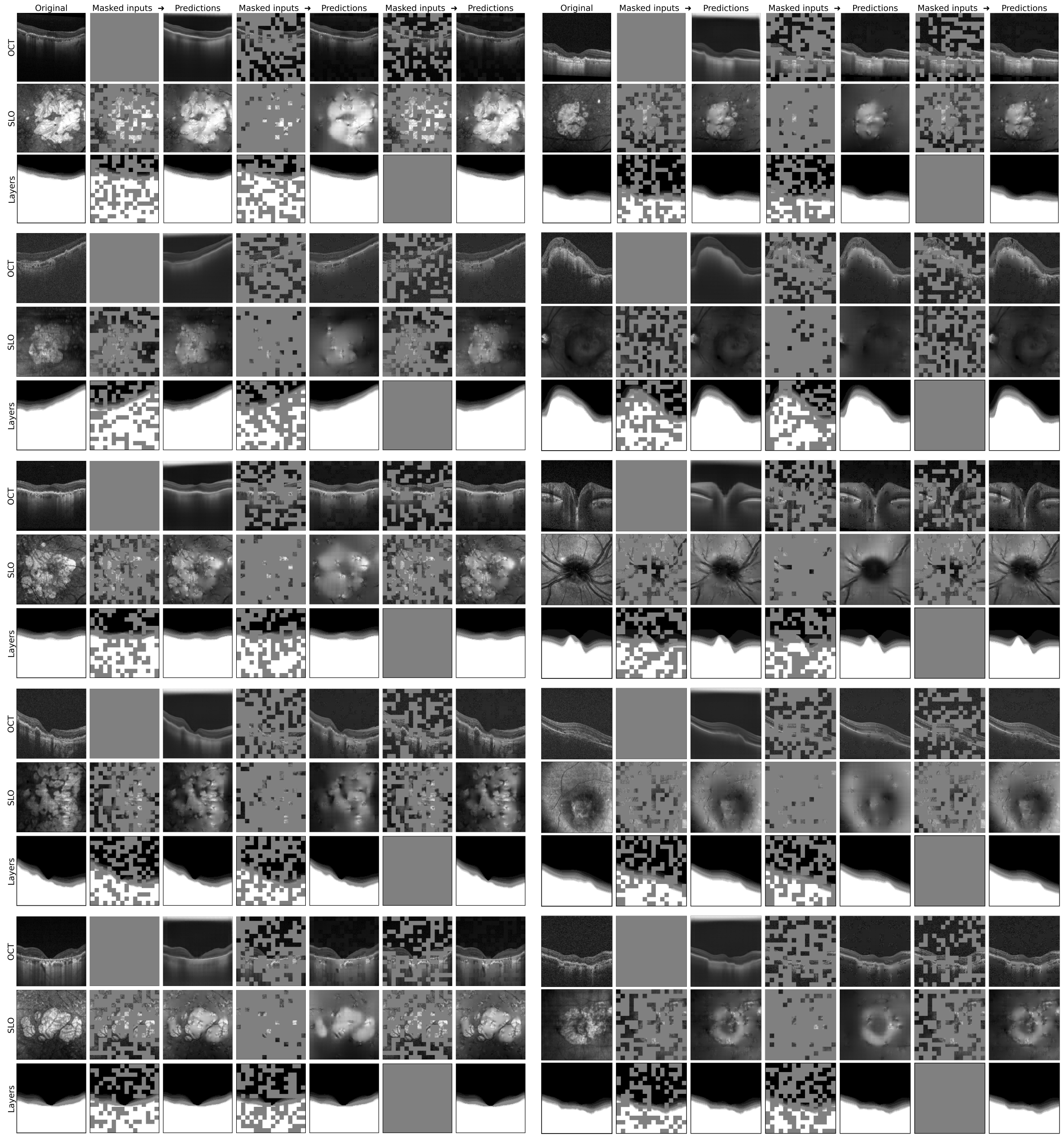

Supplementary Figure 5: MIRAGE predicitons on our internal dataset. For each sample (composed of an OCT B-scan, an SLO image, and a layer segmentation) we show the predictions with different masking proportions for each modality. The visualizations show that the model is able to reconstruct masked patches from the different modalities, suggesting that the learned representations encode meaningful multimodal information.

OCTID and Kermany datasets. Both models were used out-of-the-box without any fine-tuning. To project the embeddings into a 2D space, we used the UMAP algorithm[40]. The results are shown in Supplementary Figure 6. As shown in the figure, the embeddings produced by MIRAGE are well separated and roughly cluster the samples according to their classes. While the embeddings produced by DINOv2 and RETFound also show good separation for OCTID, they are less distinct for the Kermany dataset. This indicates that MIRAGE has learned meaningful data representations during the pretraining stage.

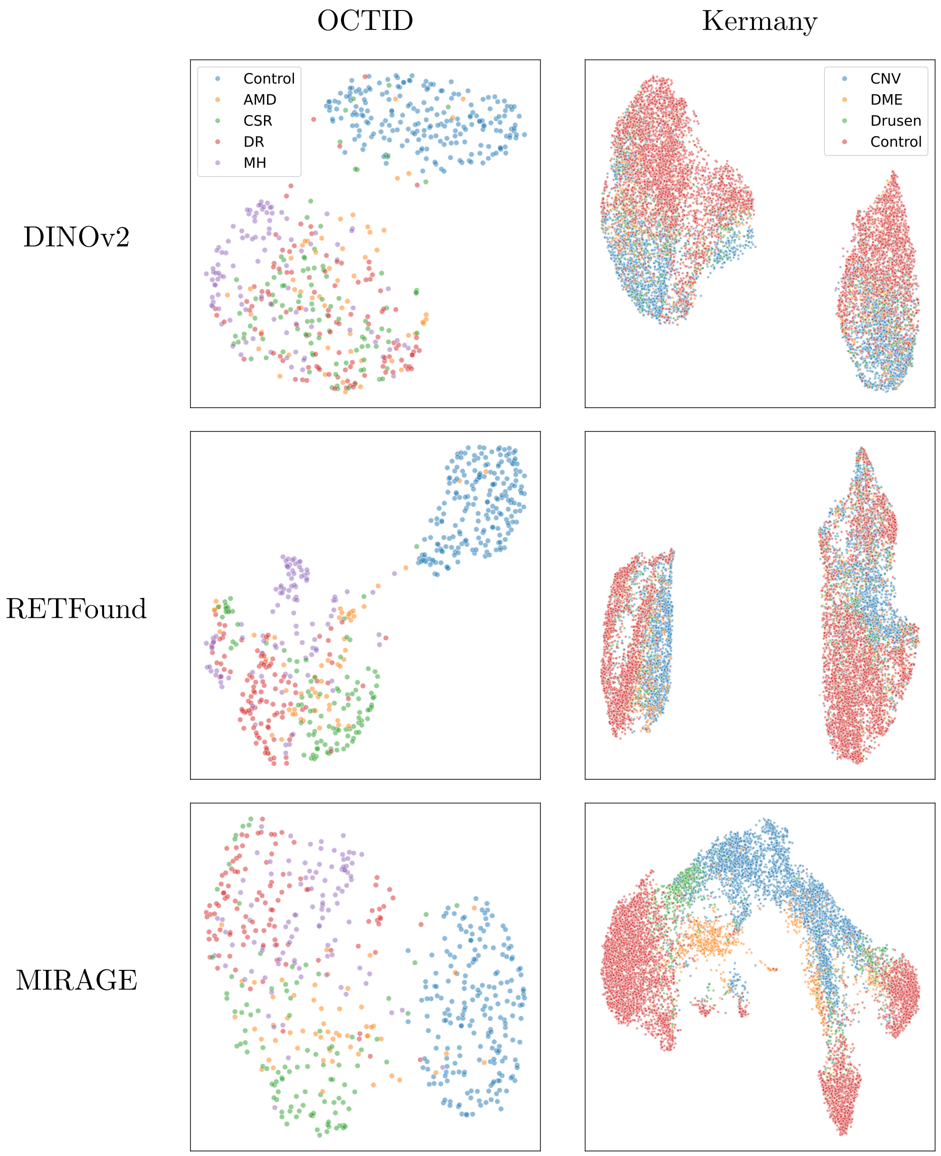

Supplementary Figure 6: Visualization of feature embeddings from different foundation models. These plots show the embeddings produced by DINOv2, RETFound, and MIRAGE for the OCTID and Kermany datasets after projection to 2D using UMAP. MIRAGE embeddings show better separation and clustering of samples compared to DINOv2 and RETFound, suggesting better representation learning.

Supplementary Note 5: Computational efficiency

To evaluate the computational efficiency of the proposed MIRAGE model, we provide, in Supplementary Table 14, the number of parameters, FLOPs, and Mult-Adds operations for the different configurations of the model, i.e., for pretraining (with three modality-specific linear projection layers [LPL] and Transformer-based decoders), for classification (with only one LPL and a linear probing head) and for segmentation (with one LPL and a ConvNeXt-based segmentation head). Both classification and segmentation configurations are common to all the adapted foundation models, which also share the same encoder architecture. The only difference is the encoder size. Thus, for RETFound and DINOv2, that use a ViT-Large encoder, the Large configuration is used, and for MedSAM, which uses ViT-Base, the Base.

In addition, we provide, in Supplementary Table 15, the computational resources and amount of data required to pretrain MIRAGE compared to other foundation models. In particular, we show the training time, number of epochs, batch size, samples and learning approach used for pretraining along with the hardware. All models except MedSAM and MultiMAE, which use ViT-Base, use ViT-Large encoders. As shown in the table, MIRAGE requires fewer computational resources and less data compared to other medical foundation models such as RETFound and MedSAM.

Supplementary Table 14: Computational efficiency of models for pretraining, classification, and segmentation tasks. The table shows the number of parameters, FLOPs and Mult-Adds operations of the models. While the values for pretraining are only for MIRAGE, the values for classification and segmentation are the same for all adapted foundation models, depending on the task and the size of the encoder. For example, RETFound and DINOv2 use a ViT-Large encoder, so the Large configuration is used. In contrast, MedSAM uses a ViT-Base encoder, so the Base configuration is used. Input size is fixed to 512×512 for the calculations.

Configuration	Size	# Parameters (M)	FLOPs (G)	Mult-Adds (G)
Pretraining	Large	318.23	236.85	1.92
	Base	99.01	69.26	1.30
Classification	Large	309.40	155.89	1.14
	Base	90.37	44.11	0.57
Segmentation	Large	315.52	396.08	2.71
	Base	96.16	171.56	2.22

Supplementary Table 15: Computational resources and data requirements for pretraining MIRAGE compared to other foundation models. The table shows the training time, the number of epochs, the batch size, the number of samples, the data type and the learning method (fully supervised [FS] or self-supervised [SS]) used for pretraining along with the used hardware.

Method	Training time	# Epochs	Batch size	# Samples	Data	Method	Hardware
SL-IN	23 days	90	4096	14.2M	RGB natural images, Classes	FS	10x TPUv3
DINOv2	>41 days	-	2048	142M	RGB natural images	SS	160x V100 (32GB)
MultiMAE	-	1600	2048	1.28M	RGB, Depth, Seg. masks	SS	8x A100 (80GB)
MedSAM	-	150	160	1.57M	10 modalities, Seg. masks	FS	20x A100 (80GB)
RETFound	14 days	800	1792	736k	OCT	SS	8x A100 (40GB)
MIRAGE	25 days	1600	256	261k	OCT, SLO, Layers	SS	1x A100 (80GB)

References

[1] Zhou, Y. *et al.* A foundation model for generalizable disease detection from retinal images. *Nature* **622**, 156–163 (2023).

[2] Oquab, M. *et al.* DINOv2: Learning robust visual features without supervision. *Transactions on Machine Learning Research* (2024).

[3] Dosovitskiy, A. *et al.* An image is worth 16x16 words: Transformers for image recognition at scale. In *Proceedings of the International Conference on Learning Representations* (2021).

[4] Isensee, F., Jaeger, P. F., Kohl, S. A. A., Petersen, J. & Maier-Hein, K. H. nnU-Net: A self-configuring method for deep learning-based biomedical image segmentation. *Nature Methods* **18**, 203–211 (2021).

[5] Bachmann, R., Mizrahi, D., Atanov, A. & Zamir, A. MultiMAE: Multi-modal multi-task masked autoencoders. In *Proceedings of the European Conference on Computer Vision*, 348–367 (Springer, 2022).

[6] Prabhushankar, M. *et al.* OLIVES dataset: Ophthalmic labels for investigating visual eye semantics. In *Advances in Neural Information Processing Systems* (2022). URL `https://zenodo.org/records/7105232`.

[7] Strudel, R., Garcia, R., Laptev, I. & Schmid, C. Segmenter: Transformer for semantic segmentation. In *Proceedings of the IEEE/CVF international conference on computer vision*, 7262–7272 (2021).

[8] Ranftl, R., Bochkovskiy, A. & Koltun, V. Vision transformers for dense prediction. In *Proceedings of the IEEE/CVF international conference on computer vision*, 12179–12188 (2021).

[9] Liu, Z. *et al.* A convnet for the 2020s. In *Proceedings of the IEEE/CVF Conference on Computer Vision and Pattern Recognition*, 11976–11986 (2022).

[10] Ma, J. *et al.* Segment anything in medical images. *Nature Communications* **15**, 654 (2024).

[11] He, Y. *et al.* SwinUNETR-V2: Stronger swin transformers with stagewise convolutions for 3D medical image segmentation. In *International Conference on Medical Image Computing and Computer-Assisted Intervention*, 416–426 (Springer, 2023).

[12] Roy, S. *et al.* MedNeXt: Transformer-driven scaling of convnets for medical image segmentation. In *International Conference on Medical Image Computing and Computer-Assisted Intervention*, 405–415 (Springer, 2023).

[13] Chen, J. *et al.* TransUNet: Rethinking the U-Net architecture design for medical image segmentation through the lens of transformers. *Medical Image Analysis* **97**, 103280 (2024).

[14] Gerendas, B. S. *et al.* Validation of an automated fluid algorithm on real-world data of neovascular age-related macular degeneration over five years. *RETINA* **42** (2022).

[15] He, K. *et al.* Masked autoencoders are scalable vision learners. In *Proceedings of the IEEE/CVF Conference on Computer Vision and Pattern Recognition*, 15979–15988 (2022).

[16] Farsiu, S. *et al.* Quantitative classification of eyes with and without intermediate age-related macular degeneration using optical coherence tomography. *Ophthalmology* **121**, 162–172 (2014). URL `https://people.duke.edu/~sf59/RPEDC_Ophth_2013_dataset.htm`.

[17] Srinivasan, P. P. *et al.* Fully automated detection of diabetic macular edema and dry age-related macular degeneration from optical coherence tomography images. *Biomedical Optics Express* **5**, 3568–3577 (2014). URL https://people.duke.edu/~sf59/Srinivasan_BOE_2014_dataset.htm.

[18] Wu, J. *et al.* GAMMA challenge: Glaucoma grading from multi-modality images. *Medical Image Analysis* **90**, 102938 (2023). URL https://gamma.grand-challenge.org/.

[19] Luo, Y., Shi, M., Tian, Y., Elze, T. & Wang, M. Harvard glaucoma detection and progression: A multimodal multitask dataset and generalization-reinforced semi-supervised learning. In *Proceedings of the IEEE/CVF International Conference on Computer Vision*, 20471–20482 (2023). URL https://ophai.hms.harvard.edu/datasets/harvard-gdp1000/.

[20] Kermany, D. Labeled optical coherence tomography (OCT) and chest X-ray images for classification (2018). URL https://data.mendeley.com/datasets/rscbjbr9sj/2.

[21] Kermany, D. S. *et al.* Identifying medical diagnoses and treatable diseases by image-based deep learning. *Cell* **172**, 1122–1131.e9 (2018).

[22] Rasti, R., Rabbani, H., Mehridehnavi, A. & Hajizadeh, F. Macular OCT classification using a multi-scale convolutional neural network ensemble. *IEEE Transactions on Medical Imaging* **37**, 1024–1034 (2017). URL https://hrabbani.site123.me/available-datasets/dataset-for-oct-classification-50-normal-48-amd-50-dme.

[23] Kulyabin, M. *et al.* OCTDL: Optical coherence tomography dataset for image-based deep learning methods. *Scientific Data* **11**, 365 (2024). URL https://dx.doi.org/10.21227/fpvs-8n55.

[24] Gholami, P., Roy, P., Parthasarathy, M. K. & Lakshminarayanan, V. OCTID: Optical coherence tomography image database. *Computers & Electrical Engineering* **81**, 106532 (2020). URL https://borealisdata.ca/dataverse/OCTID.

[25] Yu, H. J. *et al.* Real-time photographic- and fluorescein angiographic-guided management of diabetic retinopathy: Randomized PRIME trial outcomes. *American Journal of Ophthalmology* **226**, 126–136 (2021).

[26] Payne, J. F. *et al.* Randomized trial of treat and extend Ranibizumab with and without navigated laser for diabetic macular edema: TREX-DME 1 year outcomes. *Ophthalmology* **124**, 74–81 (2017).

[27] Rashno, A. *et al.* Fully automated segmentation of fluid/cyst regions in optical coherence tomography images with diabetic macular edema using neutrosophic sets and graph algorithms. *IEEE Transactions on Biomedical Engineering* **65**, 989–1001 (2018). URL https://people.ece.umn.edu/users/parhi/.DATA/.

[28] Oghbaie, M., Araújo, T., Schmidt-Erfurth, U. & Bogunović, H. VLFATRollout: Fully transformer-based classifier for retinal OCT volumes. *Computerized Medical Imaging and Graphics* **118**, 102452 (2024).

[29] Schlanitz, F. G. *et al.* Drusen volume development over time and its relevance to the course of age-related macular degeneration. *British Journal of Ophthalmology* **101**, 198–203 (2017).

[30] Bui, P. T. A. *et al.* Fundus autofluorescence and optical coherence tomography biomarkers associated with the progression of geographic atrophy secondary to age-related macular degeneration. *Eye* **36**, 2013–2019 (2022).

[31] Ritter, M. *et al.* Deep learning based quantification of photoreceptor and retinal pigment epithelium degeneration as predictive factors in stargardt disease. *Investigative Ophthalmology & Visual Science* **65**, 3777–3777 (2024).

[32] Roy, A. G. *et al.* ReLayNet: Retinal layer and fluid segmentation of macular optical coherence tomography using fully convolutional networks. *Biomedical Optics Express* **8**, 3627–3642 (2017).

[33] He, Y. *et al.* Structured layer surface segmentation for retina OCT using fully convolutional regression networks. *Medical Image Analysis* **68**, 101856 (2021).

[34] Fazekas, B. *et al.* Segmentation of Bruch's membrane in retinal OCT with AMD using anatomical priors and uncertainty quantification. *IEEE Journal of Biomedical and Health Informatics* **27**, 41–52 (2023).

[35] Melinščak, M., Radmilovič, M., Vatavuk, Z. & Lončarić, S. AROI: Annotated retinal OCT images database. In *Proceedings of the International Convention on Information, Communication and Electronic Technology*, 371–376 (2021). URL `https://ipg.fer.hr/ipg/resources/oct_image_database`.

[36] Chiu, S. J. *et al.* Kernel regression based segmentation of optical coherence tomography images with diabetic macular edema. *Biomedical Optics Express* **6**, 1172–1194 (2015). URL `https://people.duke.edu/~sf59/Chiu_BOE_2014_dataset.htm`.

[37] Fang, H. *et al.* Dataset and evaluation algorithm design for GOALS challenge. In Antony, B. *et al.* (eds.) *Ophthalmic Medical Image Analysis*, 135–142 (Springer International Publishing, Cham, 2022).

[38] Bogunović, H. *et al.* RETOUCH: The retinal OCT fluid detection and segmentation benchmark and challenge. *IEEE Transactions on Medical Imaging* **38**, 1858–1874 (2019). URL `https://retouch.grand-challenge.org/`.

[39] Arikan, M., Sadeghipour, A., Gerendas, B., Told, R. & Schmidt-Erfurt, U. Deep learning based multi-modal registration for retinal imaging. In Suzuki, K. *et al.* (eds.) *Interpretability of Machine Intelligence in Medical Image Computing and Multimodal Learning for Clinical Decision Support*, 75–82 (Springer International Publishing, Cham, 2019).

[40] McInnes, L., Healy, J. & Melville, J. Umap: Uniform manifold approximation and projection for dimension reduction. *arXiv preprint arXiv:1802.03426* (2018).
